# Prediction of Resilient Modulus Value of Cohesive and Non-Cohesive Soils Using Artificial Neural Network

**DOI:** 10.3390/ma17215200

**Published:** 2024-10-25

**Authors:** Andrzej Głuchowski

**Affiliations:** Institute of Civil Engineering, Warsaw University of Life Sciences, Nowoursynowska 166, 02-787 Warszawa, Poland; andrzej_gluchowski@sggw.edu.pl

**Keywords:** resilient modulus, artificial neural networks (ANN), pavement design, subgrade soils, subbase soils

## Abstract

This paper investigates the application of Artificial Neural Networks (ANNs) for predicting the resilient modulus (*M_r_*) of subgrade and subbase soils, which is a critical parameter in pavement design. Utilizing a dataset of 1683 *M_r_* observations, the ANN model incorporates eight input variables, including soil gradation, plasticity, and stress conditions. The model was optimized using a quasi-Newton method, achieving high predictive accuracy, with a coefficient of determination (R^2^) of 0.9613 and low error rates for both selection and testing datasets. To further enhance model interpretability, SHAP (SHapley Additive exPlanations) analysis was conducted, revealing the significant influence of specific input parameters, such as saturation ratio, plasticity index and soil gradation, on *M_r_* predictions. This study underscores the potential of ANNs as a practical tool for estimating resilient modulus, offering a reliable alternative to conventional laboratory testing methods. The findings suggest that integrating ANNs into pavement design processes can lead to more accurate predictions of pavement performance, ultimately supporting the development of more efficient and durable road structures.

## 1. Introduction

Variations in subgrade soil along road alignments present significant challenges for pavement design. To meet strict road settlement requirements, the subbase thickness must be adjusted according to subgrade conditions. Excessive settlement and deformation can be mitigated by designing proper thicknesses for hot-mix asphalt (HMA) and base course layers, ensuring that each layer supports multiple load repetitions from traffic. The goal is to distribute loads over larger areas, minimizing the risk of unwanted settlement. When accounting for environmental factors, such as temperature fluctuations and moisture changes, the task of optimizing subbase thickness becomes even more complex.

To quantify a material’s mechanical properties, designers need reliable parameters that characterize its response to cyclic loading. The road design that relies on the mechanistic–empirical models requires an elastic modulus that describes the soil material response under cyclic loading [1]. Such a parameter is the resilient modulus (*M_r_*), which is expressed as a ratio between deviator stress applied on the road layer (*σ_d_*) to the resilient axial strain (*ε_r_*) recovered after unloading.

In recent decades, road design has evolved from purely empirical methods to mechanistic–empirical approaches with the resilient modulus emerging as a critical design parameter. The methods for estimating *M_r_* have similarly progressed, shifting from empirical approaches like the California Bearing Ratio (CBR) test [2] to cyclic CBR (cCBR) [3,4] and cyclic triaxial tests [5], which provide more precise *M_r_* values. These advancements have led to the development of resilient modulus databases for various soil types. Alongside this, mathematical models to predict resilient modulus, such as those proposed by Moossazadeh and Witczak [6], Uzan [7], and Ni et al. [8], have been developed. These models often require determining specific constants (denoted as *k_i_*), derived from cyclic triaxial tests, and are influenced by factors such as confining pressure (*σ’_3_*) and deviatoric stress (*σ_d_*) [9,10,11,12,13].

The fundamental factors influencing the resilient modulus value are effective confining pressure *σ’_3_* and cyclic deviatoric stress *σ_d_* and derived stress parameters, the bulk stress *θ*, and octahedral shear stress recommended by the MEPDG [14]. Stress state parameters are the basis for resilient modulus models. Due to a very high degree of friction between the particles, the confining pressure affects non-cohesive soils more than cohesive ones [15,16,17].

Soil compaction during construction is typically achieved at the soil’s optimum moisture content (OMC) to ensure the maximum dry unit weight (MDD). The resilient modulus is highly sensitive to moisture and suction changes, which occur seasonally [18]. Environmental factors such as soil fabric, structure, and consistency also affect *M_r_* values [19].

Seasonal moisture variations significantly impact the resilient modulus of subgrade soils, particularly in fine-grained soils. Moisture content fluctuations, including dry–wet and freeze–thaw cycles, reduce the resilient modulus with the most rapid degradation occurring during the initial cycles. The impact of moisture variations is particularly critical in cold regions, where freeze–thaw cycles further degrade soil stiffness and reduce pavement fatigue life [20,21,22,23,24].

It is often pointed out that the resilient modulus of granular materials cannot be modeled solely as a function of stress [25]. In unsaturated soils, effective stress during cyclic loading does not result in significant pore pressure increase due to the dilative behavior of coarse-grained soils and low saturation levels [26]. Therefore, the saturation ratio *S_r_* plays pivotal role in *M_r_* modeling. Laboratory tests indicate that increased matrix suction raises the resilient modulus, but for fine-grained soils nearing full saturation, a sharp decline in resilient modulus occurs due to low matrix suction [27]. The compaction degree impact on the soil–water characteristic curve (SWCC) for low liquid limit clay reveals that higher water content leads to decreased compaction and significantly affects the air-entry value and water storage capacity [28]. The Plasticity Index (PI) is a key parameter in predicting resilient modulus changes in cohesive soils, especially under varying saturation conditions [29]. Therefore, the moisture or suction parameter is required to predict the resilient response of soil in seasonal changes [30].

Several models have been developed to predict resilient modulus, but many are limited by specific laboratory conditions and lack general applicability across varying soil types and conditions. Traditional nonlinear regression models, though widely used, often show poor accuracy and are restricted to specific soil types (e.g., silty, clayey, or sandy soils). To overcome these limitations, Artificial Neural Networks (ANNs) have been increasingly used to model the complex, nonlinear relationships between soil properties and resilient modulus.

ANNs offer significant advantages in modeling complex systems like soil behavior. They have been employed in various studies to predict resilient modulus with improved accuracy compared to traditional regression models [31]. For example, Saha et al. [32] developed a three-layered ANN model using 779 test results to predict *k*-constants for unbound granular base materials and plastic soils based on physical properties such as gradation, plasticity index, and moisture content. The ANN model outperformed existing regression models in terms of accuracy.

The ANN and genetic algorithm models were used to study the possibility and effect of stabilizing very weak subgrade soils at high moisture content. The input set comprised 125 sample data with eight parameters: cement and lime content, PI, silt, clay and fly ash percentage, and OMC. The developed ANN model with one hidden layer and nine nodes proved to be a reliable method to predict the resilient modulus [33].

ANNs can be used with other solutions, such as multi-population genetic algorithms, to determine the resilient modulus of compacted subgrade soils. The developed optimized ANN consisted of 10 input variables with 21 hidden neurons. The input parameters consist of physical properties: liquid limit, plastic limit, plasticity index, 0.075 mm passing percentage, MMD, and OMC; state variables: degree of compaction and moisture content; and stress variables: confining pressure and deviatoric stress. According to the analysis, the ANN estimation accuracy positively correlates with the number of datasets. The most important parameters were the physical parameters, which were followed by the state parameters and stress parameters [34].

The ANN model can be used as an alternative for Level 1 (Level 1 requires input from an actual *M_r_* test for the highest level of reliability) reliability resilient modulus estimation. The tests on nine different soils from Georgia (USA) (27 samples, training set contained 195 observations and testing set contained 75 observations) conducted in various moisture conditions and compaction levels were utilized to derive an ANN model and to determine the regression constants k of a nonlinear resilient modulus model. The developed ANN model had a higher coefficient of determination compared to the nonlinear model, and the authors denoted that the developed ANN model has the potential to reasonably estimate the subgrade resilient modulus based on physical properties and the stress state [35].

In this study, an ANN model is developed to predict the resilient modulus of subgrade soils. A range of physical and stress-related parameters is incorporated to provide accurate predictions across various soil types and soil physical properties. The originality of this work is demonstrated through a detailed exploration of the predictive capabilities of ANN for its resilient modulus and its potential application in mechanistic–empirical pavement design. The performance of the ANN model is also compared with other modern techniques to highlight its advantages in this context.

## 2. Material Properties and Objectives

### 2.1. Data Extraction

A total of 1683 resilient modulus (*M_r_*) observations were extracted from a variety of literature sources, representing a diverse dataset of subbase and subgrade soils. Eight input parameters were identified as predictors in the model:

- Percent passing the No. 200 sieve—75 µm (#200), percent passing the No. 40 sieve—425 μm (#40) is a result of soil sieve analysis;
- Plasticity Index (PI)—a measure of the plasticity of soil which is a range of moisture content over which the soil exhibits plastic behavior, indicating its ability to deform without cracking or changing volume, which is calculated as the difference between its liquid limit (LL) and plastic limit (PL);
- Dry unit weight (DUW)—the weight of soil per unit volume when the soil is completely dry. DUW helps assess soil compaction, stability, and bearing capacity;
- Void ratio (*e*)—a measure of the volume of voids (spaces between soil particles) in a soil sample relative to the volume of solid soil particles. A higher void ratio indicates a greater proportion of voids, leading to lower density and potentially weaker soil,
- Saturation ratio (*S_r_)*—a measure of the degree to which the voids in a soil sample are filled with water. *S_r_* of 0.0 indicates completely dry soil, while *S_r_* = 1.0 signifies fully saturated soil;
- Deviator stress (*σ_d_*)—the difference between the axial stress and the confining pressure acting on a soil sample. It represents the stress that causes shear deformation;
- Confining pressure (*σ’_3_*)—the pressure applied to the soil sample in the lateral direction, effectively simulating the stress conditions that exist in the ground.

The final dataset of 1683 observations exceeded the minimum requirement based on the general rule of thumb for machine learning predictions, which suggests that the sample size should be at least 50 times the number of predictors. For eight predictors, a sample size of at least 400 observations is required, but this study far exceeds that, ensuring statistical robustness. Table 1 provides a detailed picture of the characteristics of the subgrade and subbase soils used in this study.

**Figure 1:**
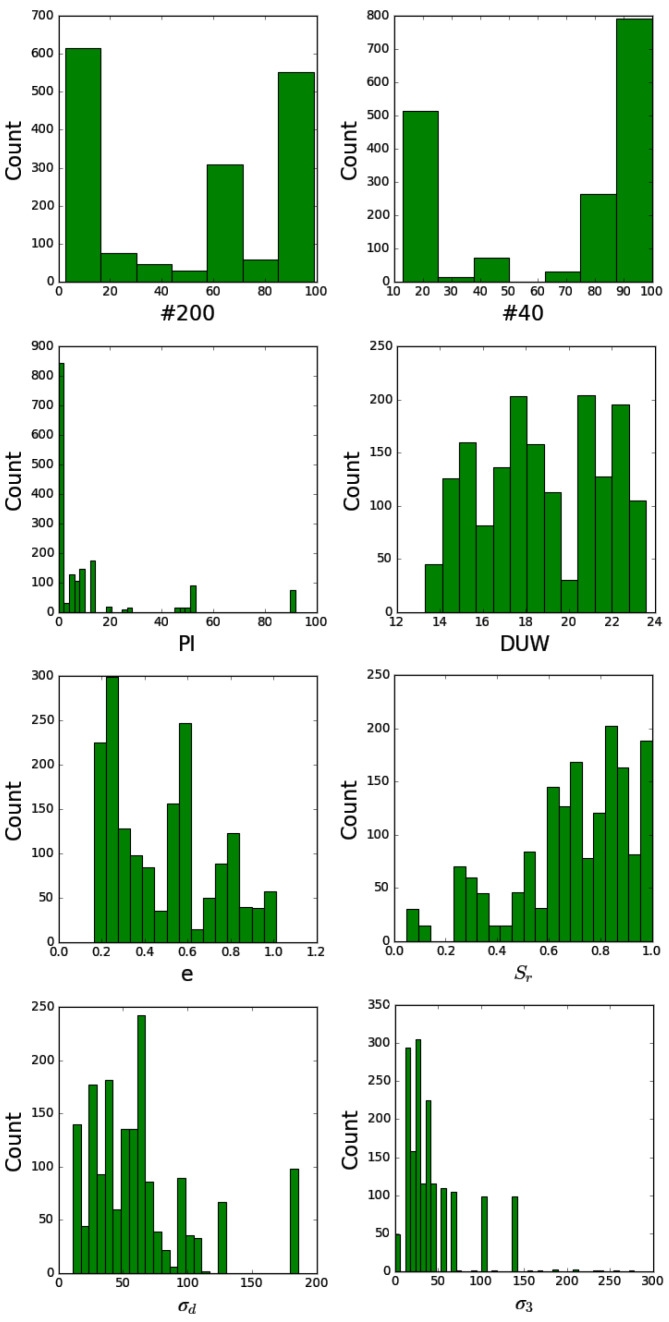
Histograms of the dataset inputs. The relationship between the number and width of bins is with respect to the Freedman–Diaconis rule.

The collected data span a wide range of soil types, which were classified according to AASHTO standards. Each row in Table 1 pertains to the average physical state for certain soil types according to AASHTO classification. Table 2 presents summary statistics (minimum, maximum, mean, and standard deviation) for each variable, and the Pearson correlation coefficients between input and output variables are displayed in Table 3. Figure 1 provides histograms of the input variables, following the Freedman–Diaconis rule to determine the number of bins, ensuring that the distribution of the input data is accurately represented.

### 2.2. Artificial Neural Network

An Artificial Neural Network (ANN) is a biologically inspired computational schema in the form of a network proposed by McCulloch and Pitts [44]. ANNs were selected due to their proven ability to handle complex, nonlinear relationships between input and output variables. ANNs have been particularly effective in cases where traditional regression models struggle with the accuracy and flexibility required for resilient modulus prediction. The feedforward multilayer perceptron (MLP) architecture was used in this study, where the information flows in one direction—from the input layer, through the hidden layers, to the output layer.

The ANN model was implemented using Neural Designer software Version 4.2.0. A total of eight input variables were fed into the network to predict the resilient modulus (*M_r_*). The hyperbolic tangent activation function was used for the hidden layers due to its nonlinear nature and symmetric output range of [−1, 1], which is advantageous in capturing the varying effects of both positive and negative inputs.

The hyperbolic tangent (*tanh*) function was chosen over other alternatives like ReLU for several reasons: (i) Symmetry: The tanh function maps inputs to an output range of [−1, 1]. In this dataset, some variables (such as *σ_d_* and *σ’_3_*) could have complex interactions, including negative influences on the *M_r_*, which the tanh function can capture effectively. (ii) Data Range: Since the input variables represent normalized soil properties, such as Plasticity Index and sieve percentages, the *tanh* function is performing better; thus, when handling inputs, normalized data are required; (iii) Model Complexity: Although ReLU is more computationally efficient and generally faster to train, it can suffer from the “dying ReLU” problem, where neurons stop learning when their output is zero for negative inputs [45].

### 2.3. ANN Model Architecture and Training

The ANN architecture consisted of three layers: an input layer with eight neurons (one for each predictor) with the min–max unsealing method, two hidden layers, and an output layer with a single neuron (for the resilient modulus prediction). The number of neurons in each hidden layer was determined through trial and error, testing models with 10 to 20 neurons per layer. The hyperbolic tangent *tanh* activation function was applied in the hidden layers. The output layer used a linear activation function, as the predicted resilient modulus can take on a wide range of values that are both positive and negative.

Training was performed using backpropagation with the Levenberg–Marquardt optimization algorithm, which is well suited for problems of this scale and complexity. The model’s performance was assessed based on the mean squared error (MSE) and coefficient of determination (R^2^).

### 2.4. Model Evaluation and Sensitivity Analysis

To ensure the robustness of the ANN model, the SHAP (SHapley Additive exPlanations) method was used for sensitivity analysis. SHAP values were calculated using Python libraries such as *shap* and *matplotlib* to evaluate the contribution of each input parameter to the predicted *M_r_*. This approach provided a clear breakdown of how each input feature influenced the model’s predictions, allowing for a comprehensive understanding of the model’s behavior. The SHAP values were visualized to identify which input parameters had the most significant impact on *M_r_* predictions.

## 3. Results

### 3.1. ANN Modeling

The ANN is a modeling method that comprises input, hidden, and output layers. The model structure was successfully utilized to model complicated relationships using interconnected adaptive simple processing elements named (artificial neurons or nodes). The remarkable ability of ANN for pattern recognition and data classification enables engineers to solve complex problems such as resilient modulus values under different loading and state conditions. To the extent of the usefulness of the ANN model, for *M_r_* prediction, one must use easy-to-calculate soil parameters which are used for ANN development in this article.

Although this study focuses on ANN modeling for predicting resilient modulus, it is worth comparing the performance of the ANN with other widely used modern machine learning techniques such as decision trees (DTs), support vector machines (SVMs), and gradient boosting algorithms (GBMs). These algorithms are also useful for various geotechnical and soil property predictions. However, each method has its own strengths and limitations regarding *M_r_* modeling in this study.

DT models are relatively easy to interpret, but they tend to overfit the data when dealing with large, complex datasets like the one in this study. SVMs are proven to be useful for the task of modeling for data exhibiting high dimensionality. However, SVMs require substantial computational resources and are time consuming. SVMs often lack the flexibility that ANNs can offer in modeling highly nonlinear data. GBMs, including XGBoost, can achieve excellent results; however, they require significant tuning and suffer from longer training times as the complexity of the dataset increases. Additionally, GBMs often require detailed hyperparameter optimization to outperform other models, including ANN.

**Figure 2:**
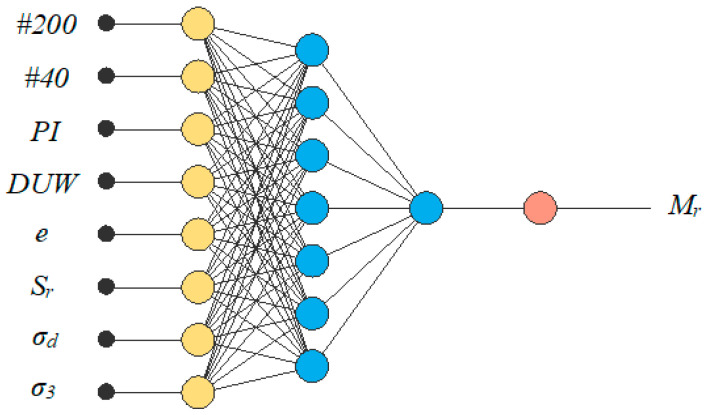
ANN final architecture (the yellow circles represent scaling neurons, the blue circles perception neurons, and the red neuron is the unscaling neuron).

The ANN architecture, in this case, consisted of three layers. The input layer was first scaled to the given data, the hidden layer, which is two layers for the first layer; the optimal order is seven neurons. An incremental order algorithm provided by the Neural Designer application was used to find the hidden layer order. The algorithm calculates the normalized squared error for different hidden layer orders. In this case, the optimum training error equal to 0.0315 was calculated for an order equal to seven. Figure 2 presents the graphical representation of the final ANN architecture. For training the ANN, the data were divided into three categories. The first was a training dataset, which constituted 60% of all data; the selection and testing instances had a 20% share of all databases. The training of the ANN model was a procedure that relied on such a training strategy that led to the best possible loss with the quasi-Newton method as an optimization algorithm. In this ANN model, as an activation function, the hyperbolic tangent *tanh* function was utilized in the first layer and the linear function was utilized for the second layer.

The ANN model first scales the input data with the mean-standard deviation method where the scaled input is equal to (1):

(1) $$I_{S}=\frac{I_{U}-\bar{x}}{\sigma_{x}}$$

where *I_U_* is an unscaled input value. The scaled input is then transferred to the hidden layer, which is the sigmoid function, but in the opposite, to the logistic function, the hyperbolic tangent function gives the output in a range from −1 to 1, and the hyperbolic tangent function is presented below (2):

(2) $$tanh(x)=\frac{e^{x}-e^{-x}}{e^{x}+e^{-x}}$$

where the output range tends to be more convenient for ANN training. The second hidden layer consists of one neuron whose activation function is linear.

For the ANN training with the use of the quasi-Newton method, we adopted the stopping criterion of the Gradient Normalization for Adaptive Loss goal in which the normalized squared error loss let the training stop after 561 epochs (final gradient equal to 0.000872). The final training normalized squared error was equal to 0.0334 and the selection normalized squared error was equal to 0.043. Equations (3) to (18) provide the final resilient modulus ANN model.

(3) $${\#200}_{s}=\frac{\#200-49.72}{37.07}$$

(4) $${\#40}_{s}=\frac{\#40-67.48}{36.24}$$

(5) $${PI}_{s}=\frac{PI-12.06}{22.11}$$

(6) $${DUW}_{s}=2\cdot \left(\frac{DUW-13.32}{23.59-13.32}\right)-1$$

(7) $$e_{s}=\frac{e-0.478}{0.238}$$

(8) $$S_{r,S}=\frac{S_{r}-0.698}{0.226}$$

(9) $$\sigma_{d,S}=\frac{\sigma_{d}-61.34}{41.33}$$

(10) $$\sigma_{3,S}=\frac{\sigma_{3}-43.66}{35.59}$$

(11) $$A=tanh(-3.35+\left(-0.91\cdot {\#200}_{s}\right)+1.62\cdot {\#40}_{s}+(-0.0994\cdot {PI}_{s})+2.22\cdot {DUW}_{S}+(-0.31\cdot e_{s})+(-1.44\cdot S_{r,S})+0.094\cdot \sigma_{d,S}+0.48\cdot \sigma_{3,S}))$$

(12) $$B=\tanh(5.36+\left(-2.40\cdot {\#200}_{s}\right)+0.46\cdot {\#40}_{s}+0.012\cdot {PI}_{s}+\left(-3.26\cdot {DUW}_{S}\right)+0.49\cdot e_{s}+1.05\cdot S_{r,S}+1.12\cdot \sigma_{d,S}+(-0.52\cdot \sigma_{3,S}))$$

(13) $$C=\tanh\left(0.43+0.051\cdot {\#200}_{s}+1.07\cdot {\#40}_{s}+(-1.23\cdot {PI}_{s}\right)+2.22\cdot {DUW}_{S}+1.62\cdot e_{s}+1.13\cdot S_{r,S}+0.15\cdot \sigma_{d,S}+(-0.42\cdot \sigma_{3,S}))$$

(14) $$D=\tanh(-1.63+(-1.18\cdot {\#200}_{s})+1.30\cdot {\#200}_{s}+1.02\cdot {PI}_{s}+(-0.52\cdot {DUW}_{S})+(-1.45\cdot e_{s})+0.19\cdot S_{r,S}+0.08\cdot \sigma_{d,S}+(-0.15\cdot \sigma_{3,S}))$$

(15) $$E=\tanh(0.56+(-0.44\cdot {\#200}_{s})+0.55\cdot {\#40}_{s}+0.71\cdot {PI}_{s}+(-2.81\cdot {DUW}_{S})+(-1.98\cdot e_{s})+0.11\cdot S_{r,S}+(-0.14\cdot \sigma_{d,S})+(-0.15\cdot \sigma_{3,S}))$$

(16) $$F=\tanh\left(1.43+1.24\cdot {\#200}_{s}+(-1.15\cdot {\#40}_{s})+(-0.82\cdot {PI}_{s}\right)+(-0.70\cdot {DUW}_{S})+0.77\cdot e_{s}+(-0.20\cdot S_{r,S})+(-0.12\cdot \sigma_{d,S})+0.11\cdot \sigma_{3,S})$$

(17) $$G=\tanh(0.39+(-0.12\cdot {\#200}_{s})+0.22\cdot {\#40}_{s}+0.23\cdot {PI}_{s}+(-1.07\cdot {DUW}_{S})+(-0.71\cdot e_{s})+0.01\cdot S_{r,S}+(-0.04\cdot \sigma_{d,S})+(-0.03\cdot \sigma_{3,S}))$$

(18) $$M_{r}=700\cdot \left(\left(0.75+(A\cdot 0.28)+(B\cdot -1.90)+(C\cdot -0.13)+(D\cdot -0.91)+(E\cdot -0.60)+(F\cdot -0.74)+(G\cdot 1.88)\right)+1\right)$$

The details of all errors for the ANN model are present in Table 4. The sum squared error (SSE) shows that the model has learned patterns from training data, due to the decreasing SSE for the testing set. The mean squared error (MSE) for the training set (449.6) is significantly lower than both the selection (1168.7) and testing (980.4) sets. The higher MSE in the selection and testing sets compared to the training set indicates that the model performs slightly worse on unseen data, which is expected. However, the gap between training and testing MSE is not large (449.6 vs 980.4), suggesting that the model generalizes well. The root mean squared error (RMSE) follows the same trend as the MSE. The training RMSE is lower at 21.20, while the testing RMSE is higher at 31.31. The normalized squared error (NSE) is lowest in the training set (0.034) and increases slightly in the selection (0.052) and testing (0.065) sets. The model performs slightly worse on unseen data, but this difference is small, indicating reasonable generalization. The testing NSE (0.065) being only slightly higher than the selection NSE (0.052) suggests that the model performs consistently and generalizes well across different datasets. The Minkowski error (E_Minkowski_) shows the model’s flexibility in handling different types of errors (combining absolute and squared differences). Interestingly, the Minkowski error decreases from training (78,746.4) to testing (42,154.4). This suggests that while the model is tuned for training, it has performed better on unseen data (testing set). A lower Minkowski error on the testing set reflects good generalization and indicates that the model is robust in handling both small and large errors.

It is important to note that the application of the ANN model is limited to the cases represented by the data in Figure 1. Although the data range is wide, the ANN model should be used with caution outside this range (especially when higher value of *σ_d_* and *σ’_3_* is applied).

**Figure 3:**
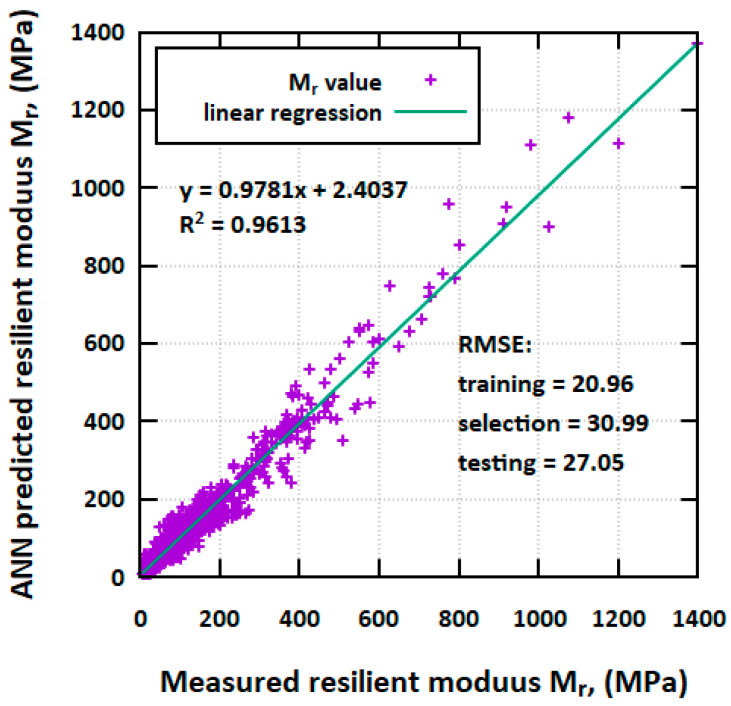
Comparison between ANN models’ prediction and measured resilient modulus *M_r_* value.

For ANN models’ prediction quality, the comparison between the measured and prognosed *M_r_* value is presented in Figure 3. The R^2^ for the deployed ANN model is equal to 0.9613, which shows the high accuracy of the model for *M_r_* prediction.

**Figure 4:**
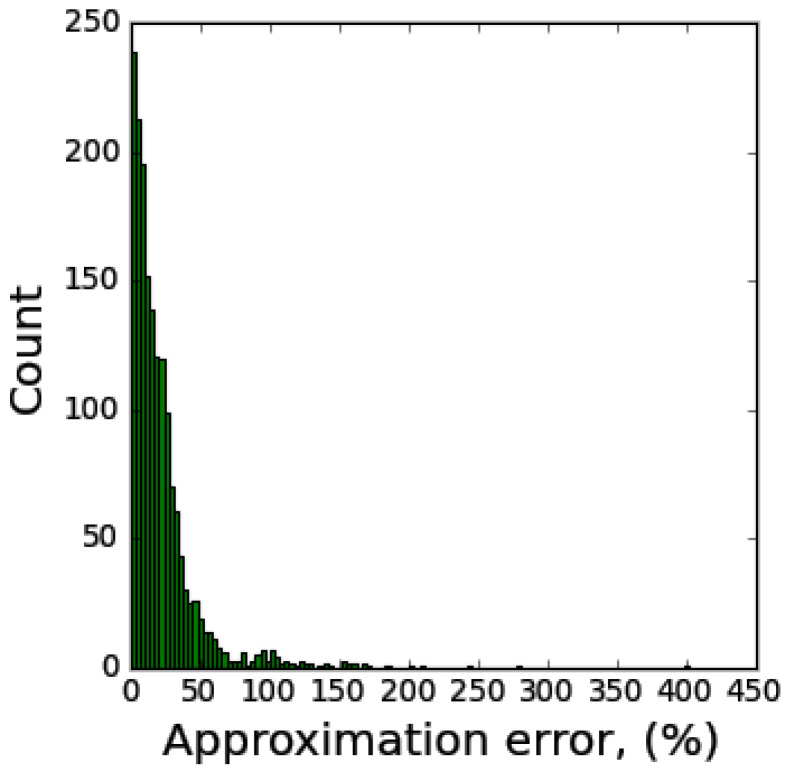
Resilient modulus approximation error histogram.

To highlight the distribution shape of the resilient modulus approximation error, a histogram is presented in Figure 4. The approximation error is calculated by the following formula (19):

(19) $$\delta =\left(\frac{v_{a}-v_{e}}{v_{e}}\right)\cdot 100\%$$

The histogram indicates that 61% of the results fall within the range of δ < 20%, while 92% of the results fall within δ < 50%. Furthermore, the outliers from the examined ANN model are limited to a few instances (fewer than 20 cases), which are characterized by low deviator stress (*σ_d_* < 20 kPa).

### 3.2. Sensitivity Analysis

**Figure 5:**
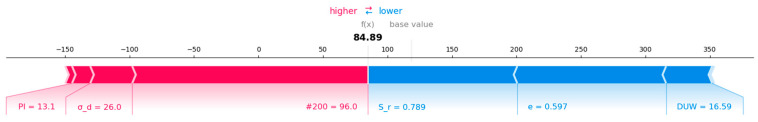
SHAP analysis of input values for deployed ANN *M_r_* model.

For the ANN model’s robustness analysis, the SHAP (SHapley Additive exPlanations) method was applied. The results provide a detailed breakdown of how each input feature influenced the model’s predictions (Figure 5).

The force plot generated from SHAP values helped to identify specific input parameters that had the most significant impact on *M_r_* predictions. Features with positive SHAP values, such as PI, *σ_d_*, and #200, contributed to increasing the predicted *M_r_*, highlighting their importance in the model’s decision-making process. Conversely, features with negative SHAP values, such as DUW, *e,* and *S_r_*, were shown to reduce the predicted *M_r_*, indicating their potential to detract from the overall prediction.

These observations not only clarify the relative importance of each feature but also underscore the complex interactions between parameters. By interpreting the SHAP values, it can be concluded that special attention is required when #200, *e,* and *S_r_* are used.

## 4. Conclusions

This paper employed Artificial Neural Network (ANN) techniques to predict the resilient modulus (*M_r_*) for cohesive and noncohesive soils. The ANN was utilized to estimate the resilient modulus across a wide range of physical properties and stress states. Resilient modulus tests were conducted using a repeated loading triaxial apparatus on 1683 samples from the literature, providing a robust dataset for model training. The ANN architecture, featuring two hidden layers—one with seven nodes and another with one node—demonstrated reliability in predicting Mr. The model was simulated using Neural Designer software Version 4.2.0, further validating its effectiveness.

The hyperbolic tangent function served as the activation function in the hidden layers, while a linear function was applied in the output layer. The optimal architecture was identified using an incremental order algorithm, confirming the selection of seven neurons in the first hidden layer. Model performance was evaluated on a dataset divided into training (60%), selection (20%), and testing (20%) sets, achieving a high coefficient of determination (R^2^ = 0.9613). The normalized squared error was low for both the selection (0.052) and testing (0.065) datasets, underscoring the model’s robustness.

A histogram of approximation errors revealed that 61% of predictions had an error of less than 20%, and 92% had an error of less than 50%, with very few outliers. These findings highlight the reliability of the ANN model across various soil types and conditions. Furthermore, sensitivity analysis using the SHAP (SHapley Additive exPlanations) method showed the contributions of input features to the model’s predictions, emphasizing the importance of parameters such as *S_r_*, *σ_d_*, and #200. The SHAP values provided a clear understanding of how each input feature influences the model’s outputs, enhancing interpretability and trust in the ANN’s decision-making process.

The study confirms that ANN models are highly effective in predicting the resilient modulus of subgrade and subbase materials, outperforming traditional nonlinear regression models. The ability of ANNs to model complex, nonlinear relationships make them useful in pavement design, particularly when addressing diverse soil types and environmental conditions. By integrating ANNs into the design process, engineers can achieve more accurate pavement performance predictions, resulting in more efficient and durable road structures. The insights gained from SHAP analysis further enable engineers to prioritize key parameters during design and assessment, ultimately leading to improved decision making in pavement engineering.

In general, the ANN model shows reasonable generalization. A drawback is that the selection set errors are higher than those in the testing set (as shown in Table 4), indicating the possibility of further ANN improvement by selecting hyperparameters. Further tuning of the model is recommended to minimize errors in the selection set. A possible solution is the application of AdaBoost techniques alongside ANN optimization.

## Figures and Tables

**Table 1:** Characteristics of subgrade and subbase soils utilized in this study.

Data Source	PI (%)	#200 (%)	#40 (%)	OMC (%)	DUW (kN/m^3^)	Classification by AASHTO
George [9] Total observations: 50	6.1	55	100	13.8	18.1	A-4
	8	56	100	14.1	17.82	A-4
	7	40	100	12.9	18.56	A-4
	12.4	60	90	13.8	18.14	A-6
	13.1	96	99	17.8	16.59	A-6
	1	28	100	11.0	18.53	A2-4
	4.9	42	100	12.0	18.67	A-4
	13.3	98	99	18.6	16.67	A-6
Li et al. [36] Total observations: 513	0	10	19.5	5.5	23.59	A-1-a
	0	8	18	5.4	22.15	A-1-a
	0	6	15.5	5.3	23.44	A-1-a
	0	3.15	13	5.3	23.06	A-1-a
Maher et al. [37] Total observations: 118	0	7.6	39	8.75	18.72	A-1-b
	3	30.1	76	8.5	19.37	A-2-4
	0	33.3	75	9.0	18.15	A-2-4
	0	9.9	83	9.0	17.37	A-3
	1.5	36.6	77	8.25	19.26	A-4
	4	43	77	8.5	19.29	A-4
	18.9	97.5	99	14.5	17.08	A-6
	27.4	97.7	99	22.5	15.67	A-7
Gupta et al. [38] Total observations: 383	11	85.3	95	13.5	17.9	A-4
	24	91.3	97	22.0	15.87	A-7-6
	9	58.6	86	16.0	17.36	A-4
	52	96.4	98	27.5	14.4	A-7-6
Han and Vanapalli [18] Total observations: 21	6	96.5	99.5	12.75	19.5	A-4
	19	77	96	13.2	19.39	A-6
	26	79.5	94	23.5	16.16	A-6
Malla and Joshi [39] Total observations: 193	0	5	35	11.2	18.41	A-1-a
	6	23.3	39	10.3	19.02	A-2-4
	6	26.9	46	11.1	18.86	A-2-5
	6	22.8	38	10.0	18.98	A-1-b
	0	3.1	79.3	14.7	15.63	A-3
	0	4	69.4	12.5	15.67	A-3
	0	2.6	75.5	13.8	15.62	A-3
	51	99	99.6	30.3	13.33	A-7-6
	47	98.3	99.4	30.5	13.32	A-7-7
	49	98.8	99.6	30.4	13.4	A-7-8
	8	81.9	94.5	14.2	17.72	A-6
	8	77.6	93.6	15.5	17.15	A-6
	8	84.3	96.8	16.1	16.76	A-6
Liang et al. [40] Total observations: 50	8	56.3	75.7	14.2	17.75	A-4-a
	12.3	68.8	81.1	16.5	17.7	A-6-a
Banerjee et al. [41] Total observations: 30	0	90	99.5	14.8	16.5	A-4
	92	95	99.9	22.9	14.4	A-7-6
Pereira et al. [42] Total observations: 16	0	3	50	8.9	21.14	A-1-b
Hanittinan [43] Total observations: 309	9	62	100	12.4	20.58	A-4
	8	64	100	12.6	18.85	A-4
	6	59	100	12.1	20.67	A-4

**Table 2:** The data statistics of the dataset in this study.

Variable *x*	Minimum min(*x*)	Maximum max(*x*)	Mean $(\bar{x}$)	$\mathrm{Standard}\;\mathrm{Deviation}\;(\sigma_{x})$
#200 (%)	2.6	99	49.72	37.07
#40 (%)	13	100	67.48	36.24
PI (%)	0.0	92	12.06	22.11
DUW (kN/m^3^)	13.32	23.59	18.7	2.87
e (–)	0.16	1.0	0.48	0.24
S_r_ (–)	0.05	1.0	0.689	0.226
σ_d_ (kPa)	11.0	186.16	61.34	41.34
σ_3_ (kPa)	0.0	278.41	43.66	35.59
M_r_ (MPa)	9.2	1400	111.07	124.25

**Table 3:** The Pearson linear correlation between input and input-target variables.

	#200	#40	PI	DUW	e	Sr	σ_d_	σ₃	M_r_
#200	1	0.89	0.56	–0.8	0.72	0.31	0.3	–0.34	–0.22
#40	0.89	1	0.44	–0.79	0.63	0.38	0.42	–0.47	–0.32
PI	0.56	0.44	1	–0.63	0.64	0.13	–0.18	–0.19	–0.21
DUW	–0.8	–0.79	–0.63	1	–0.95	0.17	0.34	0.35	0.23
e	0.72	0.63	0.64	–0.95	1	0.11	–0.21	–0.23	–0.2
Sr	0.31	0.38	0.13	–0.17	0.11	1	–0.21	–0.2	–0.66
σ_d_	0.3	0.42	–0.18	0.34	–0.21	–0.21	1	0.58	0.26
σ₃	–0.34	–0.47	–0.19	0.35	–0.23	–0.2	0.58	1	0.37
M_r_	–0.22	–0.32	–0.21	0.23	–0.2	–0.66	0.26	0.37	1

**Table 4:** Errors table for resilient modulus ANN model.

Error	Training	Selection	Testing
Sum squared error (SSE)	455,010	393,835	330,403
Mean squared error (MSE)	449.6	1168.7	980.4
Root mean squared error (RMSE)	21.20	34.19	31.31
Normalized squared error (NSE)	0.034	0.052	0.065
Minkowski error (E_Minkowski_)	78,746.4	48,206.9	42,154.4

## Data Availability

Dataset available on request from the author.
